# Reviewing the evidence supporting predictive biomarkers in European medicines agency indications and contraindications using visual plots

**DOI:** 10.1186/1745-6215-16-S2-P157

**Published:** 2015-11-16

**Authors:** Kinga Malottki, Lucinda Billingham, Richard Riley, Jonathan Deeks

**Affiliations:** 1 University of Birmingham, Birmingham, UK; 2 Keele University, Keele, UK

## Objectives

Predictive biomarkers can be used to identify most suitable treatments for patients. To maximise patient benefit valid predictive biomarkers need to be used and several trial designs have been proposed to achieve this.

We aimed to review the evidence considered sufficient by the European Medicines Agency (EMA) for inclusion of a predictive biomarker in a drug indication or contraindication and this required development of a methodology to summarise key information.

## Methods

We reviewed the evidence supporting 49 biomarker-indication-drug combinations (37 different biomarkers and 41 drugs) within EMA licensing. The main issues considered were: biomarker evaluation design, sample size, representativeness of patients in trials, primary outcome type and strength of findings. We developed a novel visual overview of these dimensions in radial plots. We explored whether there were differences in the evidence base depending on authorisation recommendation, disease area, biomarker type and orphan designation.

## Results

155 studies were identified: one biomarker strategy design, 45 enrichment studies, 37 RCT subgroup analyses and 72 other designs (mainly single arm, case series and reports). A median of 2 studies (range: none to 19) supported a biomarker-indication-drug combination. Comparison of evidence depending on factors outlined above will be reported.

## Conclusions

There was a wide variation in type and quantity of evidence supporting biomarker-indication-drug combinations and the radial plots were a useful means of assessment. Study designs used were not always robust. It was not always clear why certain predictive biomarkers were included in the indication. The role of the biomarker evaluation context seemed important.

